# Priming of innate antimycobacterial immunity by heat-killed *Listeria monocytogenes* induces sterilizing response in the adult zebrafish tuberculosis model

**DOI:** 10.1242/dmm.031658

**Published:** 2018-01-01

**Authors:** Hanna Luukinen, Milka Marjut Hammarén, Leena-Maija Vanha-aho, Aleksandra Svorjova, Laura Kantanen, Sampsa Järvinen, Bruno Vincent Luukinen, Eric Dufour, Mika Rämet, Vesa Pekka Hytönen, Mataleena Parikka

**Affiliations:** 1 Faculty of Medicine and Life Sciences, FI-33014 University of Tampere, Tampere, Finland; 2 BioMediTech Institute, FI-33014 University of Tampere, Tampere, Finland; 3 PEDEGO Research Unit, and Medical Research Center Oulu, FI-90014 University of Oulu, Oulu, Finland; 4 Department of Children and Adolescents, Oulu University Hospital, FI-90220 Oulu, Finland; 5 Fimlab Laboratories, Pirkanmaa Hospital District, FI-33520 Tampere, Finland; 6 Oral and Maxillofacial Unit, Tampere University Hospital, FI-33521 Tampere, Finland

**Keywords:** *Listeria monocytogenes*, *Mycobacterium marinum*, Mycobacterial infection, Sterilizing immunity, Tuberculosis, Zebrafish

## Abstract

Mycobacterium tuberculosis remains one of the most problematic infectious agents, owing to its highly developed mechanisms to evade host immune responses combined with the increasing emergence of antibiotic resistance. Host-directed therapies aiming to optimize immune responses to improve bacterial eradication or to limit excessive inflammation are a new strategy for the treatment of tuberculosis. In this study, we have established a zebrafish-*Mycobacterium marinum* natural host-pathogen model system to study induced protective immune responses in mycobacterial infection. We show that priming adult zebrafish with heat-killed *Listeria monocytogenes* (HKLm) at 1 day prior to *M. marinum* infection leads to significantly decreased mycobacterial loads in the infected zebrafish. Using *rag1*^−/−^ fish, we show that the protective immunity conferred by HKLm priming can be induced through innate immunity alone. At 24 h post-infection, HKLm priming leads to a significant increase in the expression levels of macrophage-expressed gene 1 (*mpeg1*), tumor necrosis factor α (*tnfa*) and nitric oxide synthase 2b (*nos2b*), whereas superoxide dismutase 2 (*sod2*) expression is downregulated, implying that HKLm priming increases the number of macrophages and boosts intracellular killing mechanisms. The protective effects of HKLm are abolished when the injected material is pretreated with nucleases or proteinase K. Importantly, HKLm priming significantly increases the frequency of clearance of *M. marinum* infection by evoking sterilizing immunity (25 vs 3.7%, *P*=0.0021). In this study, immune priming is successfully used to induce sterilizing immunity against mycobacterial infection. This model provides a promising new platform for elucidating the mechanisms underlying sterilizing immunity and to develop host-directed treatment or prevention strategies against tuberculosis.

This article has an associated First Person interview with the first author of the paper.

## Abstract (precis)

**Summary:** Heat-killed *Listeria monocytogenes* induces immune responses that lead to increased clearance of mycobacterial infection in the adult zebrafish tuberculosis model via innate immune mechanisms.

## INTRODUCTION

Tuberculosis (TB) is an airborne respiratory disease caused by the intracellular bacterium *Mycobacterium tuberculosis* (Mtb). As few as one to five bacteria can lead to an infection (Rajaram et al., 2014; Cambier et al., 2014)*.* The outcome of TB is highly variable, ranging from rapid clearance by innate immune mechanisms to development of active disease or the formation of a latent infection that can be actively contained inside granulomas but not eradicated. According to Centers for Disease Control and Prevention (CDC) estimates, even one third of the world population is infected with Mtb. However, only 5-10% of this population develops active, primary TB. Commonly, infection with Mtb leads to a latent, asymptomatic disease with the inherent ability to reactivate and disseminate into an active disease even decades after initial exposure, for example in the case of immunosuppression. In 2015, 1.4 million people died of TB and a total of 10.4 million new cases were reported along with an increasing number of multidrug-resistant strains (World Health Organization, 2016; http://www.who.int/tb/publications/2016/en/). Despite available multidrug therapies and the Bacille Calmette–Guérin (BCG) vaccine, TB remains one of the leading infectious killers worldwide. According to a recent study, the standard 6-month antibiotic treatment against TB is ineffective in the eradication of Mtb even in patients with a successful follow through of the antibiotic treatment (Malherbe et al., 2016). As the current preventive and treatment strategies have proven insufficient, new approaches to control the global TB epidemic are urgently needed. Host-directed therapies offer a promising approach to improve the outcome of anti-TB treatments. Host-directed therapies are a form of adjunctive therapy that aim to modulate the host immune responses to eradicate or limit mycobacterial infection (Tobin, 2015).

Mycobacteria are especially successful in evading immune responses. Macrophages are known to limit mycobacterial growth in early infection to some extent (Clay et al., 2007). However, in many cases, the early events of mycobacterial infections are characterized by bacterial dominance. Pathogenic mycobacteria are able to avoid recognition by pattern-recognition receptors and can lure mycobacterium-permissive macrophages to the sites of infection (Cambier et al., 2014). Upon phagocytosis, they block the fusion of phagosomes with lysosomes (Russell, 2011), translocate to the cytoplasm (Simeone et al., 2012; Houben et al., 2012) and neutralize nitric oxide (NO) species (Flynn and Chan, 2003), allowing them to survive within macrophages. Astonishingly, mycobacteria are even capable of exploiting macrophages for tissue dissemination (Clay et al., 2007). In addition to avoiding innate killing mechanisms, mycobacteria also inhibit transportation of mycobacterial antigens to lymph nodes (Wolf et al., 2008; Reiley et al., 2008; Gallegos et al., 2008), thereby hampering the initiation of adaptive responses (Chackerian et al., 2002). Aggregates of innate and adaptive immune cells, called granulomas, are formed to contain the bacteria and to localize the infection to a limited area without eradicating the bacteria. Depending on the immune status of the host, either an active infection or a latent infection with a life-long risk of reactivation ensues (Barry et al., 2009).

Despite Mtb being good at evading host immune responses and having the ability to cause aggressive active or persistent latent infections, some people are known to be naturally protected against TB. There are significant differences in the ability of individuals to resist mycobacterial infection, reflecting the heterogenic nature of the human population. According to epidemiological data, a 7-43% proportion of heavily exposed individuals are able to clear the infection before the onset of adaptive immunity, resulting in negative tuberculin skin tests and interferon-gamma (Ifnγ) release assays (reviewed in Verrall et al., 2014). With this in mind, it should be possible to shift the balance of host-pathogen interactions in favor of the host by directing the immune response to the right immune activation at the early stages of infection, when the bacterial loads are rather small. Optimal immune activation could prevent mycobacterial evasion strategies, enhance killing of mycobacteria and ideally lead to sterilization of infection. However, to our knowledge, sterilizing antimycobacterial immunity has not been successfully induced *in vivo.*

In this study, we used the zebrafish model to study protective immune responses against mycobacteria at the early stages of infection. The zebrafish has recently become a well-accepted genetically tractable vertebrate model for human TB pathogenesis. Zebrafish are naturally susceptible to *Mycobacterium marinum*, a close genetic relative of Mtb. *M. marinum* causes a disease that shares the main pathological and histological features of human TB, including the formation of macrophage aggregates and granulomas (reviewed in Meijer, 2016; van Leeuwen et al., 2014). As the basic mechanisms of innate and adaptive immunity are conserved from zebrafish to humans, the innate immune responses to *M. marinum* can be studied in zebrafish larvae (Ramakrishnan, 2013), while the adult zebrafish has been proven an applicable model to study also the adaptive responses in TB (Hammarén et al., 2014; Parikka et al., 2012; Oksanen et al., 2013). Even the transition of an acute primary infection to latency and its reactivation can be modeled in the adult zebrafish (Parikka et al., 2012), which has been difficult in other models. Our previous studies have shown that an infection with a low dose of *M. marinum* causes a latent mycobacterial disease with steady bacterial counts in the majority of the fish population, whereas, in a small proportion of the fish (<1.5%), primary active disease leads to mortality (Parikka et al., 2012). Around 10% of the fish are able to clear the mycobacterial infection (Hammarén et al., 2014). The spectrum of different disease outcomes in the *M. marinum*-zebrafish model thus resembles that of human TB.

Here, we have used the zebrafish model to test whether the number of individuals sterilizing the infection can be increased through injection with different priming agents to circumvent mycobacterial virulence strategies, which generally lead to persistent, latent infections (Parikka et al., 2012). Our study shows that the priming of zebrafish with heat-killed *Listeria monocytogenes* results in the sterilization of *M. marinum* infection in one fourth of individuals. The protective effect is caused by a protein and/or nucleic acid component of *L. monocytogenes* and is accompanied by the induction of *tumor necrosis factor α* (*tnfα*) and *nitric oxide synthase 2b* (*nos2b*), and downregulation of *superoxide dismutase 2* (*sod2*). Hereby, we show that the adult zebrafish is a feasible model for deciphering the mechanisms of sterilizing immunity, knowledge of which is crucial for the development of new preventive strategies and adjunctive therapies against TB.

## RESULTS

### Immune activation by heat-killed *L. monocytogenes* leads to lower mycobacterial burdens in adult zebrafish

**Fig. 1.**
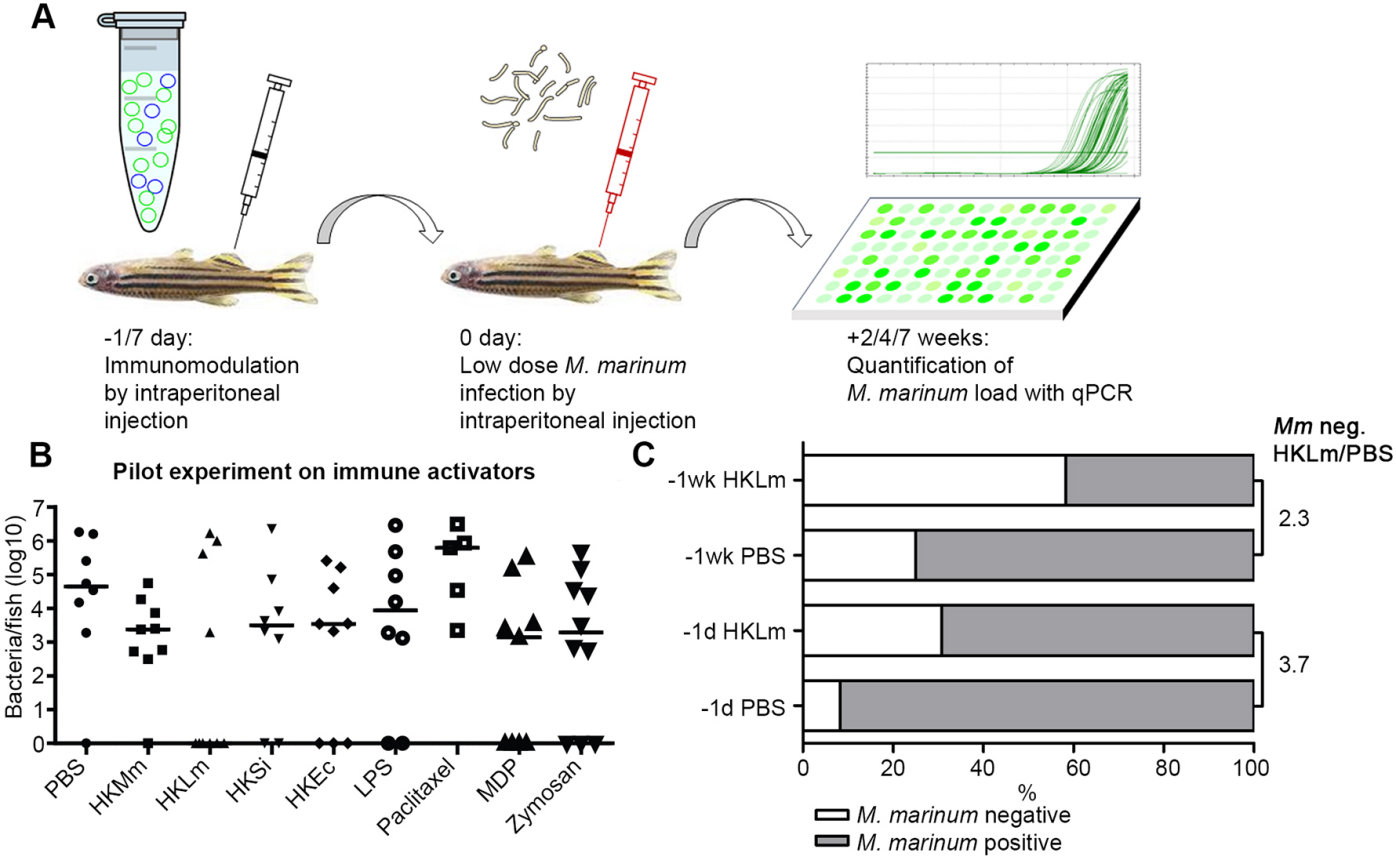
**The effect of priming agents on *M. marinum* loads in adult zebrafish.** (A) Outline of the study. Adult wild-type zebrafish were primed with 0.5×10^7^-1×10^7^ colony-forming units (cfu) per fish of heat-killed bacteria or with other priming agents (13.5 µg per fish, except MDP 4.5 µg) 1 or 7* *days prior to *M. marinum* infection (−1/7* *day) with an i.p. injection. Sterile 1× PBS was used as an injection control. The following day (0 day), a low dose of *M. marinum* was injected i.p. into the zebrafish. Internal organs were collected either 2, 4 or 7 weeks post infection (wpi), DNA was extracted and the bacterial counts were measured with *M. marinum*-specific qPCR. (B) Priming with heat-killed *L. monocytogenes* (HKLm) reduces mycobacterial loads at 7 wpi. Zebrafish were primed with either PBS (*n*=8), heat-killed *M. marinum* (HKMm; *n*=9), HKLm (*n*=10), heat-killed *S. iniae* (HKSi; *n*=8), heat-killed *E. coli* (KHEc; *n*=9), lipopolysaccharide (LPS; *n*=8), paclitaxel (*n*=5), muramyl dipeptide (MDP; *n*=9) or zymosan (*n*=10) 1* *day prior to *M. marinum* infection (16±4 cfu). Organs were collected at 7 wpi. Priming with HKLm led to a bigger decrease in the mycobacterial loads in adult zebrafish compared to other tested priming agents. Paclitaxel increased the mycobacterial loads in adult zebrafish. Medians are shown in the figure. (C) Priming with HKLm 1* *day prior to *M. marinum* infection increases the frequency of clearance. The fold change in the percentage of fish that were able to clear the *M. marinum* infection was higher in the group that was primed with HKLm 1* *day prior (3.7-fold; PBS: *n*=12, HKLm: *n*=13) to infection compared to group that was primed 7* *days (2.3-fold; PBS: *n*=12, HKLm: *n*=12) before the infection.

In this study, we set out to investigate protective immune responses at the early stages of an *M. marinum* infection. Our hypothesis is that the right immune activation at the early stages of an infection prevents mycobacterial evasion strategies and leads to protective immune responses, increased killing of mycobacteria or even clearing of mycobacteria. To study our hypothesis, we wanted to direct the zebrafish immune response towards an optimal anti-TB response before the onset of an *M. marinum* infection. To find a factor that promotes a protective immune response in the early stages of an infection in adult zebrafish, fish were intraperitoneally (i.p.) injected with different priming agents 1 day before a low-dose i.p. *M. marinum* infection (Fig. 1A,B). Mycobacterial loads of the fish were determined with quantitative real-time PCR (qPCR) from internal organs at 7 weeks post-infection (wpi). We tested eight different agents, including Toll-like receptor (TLR) ligands, vaccine adjuvants and heat-killed bacteria, namely: heat-killed *M. marinum* (HKMm), *L. monocytogenes* (HKLm), *Streptococcus iniae* (HKSi) and *Escherichia coli* (HKEc), lipopolysaccharide (LPS), paclitaxel, muramyl dipeptide (MDP) and zymosan.

In this preliminary experiment, the tested priming agents had variable effects on mycobacterial loads. Priming of the adult fish with HKLm led to a clear decrease in median mycobacterial loads at 7 wpi compared to phosphate-buffered saline (PBS) controls. However, with most of the other tested priming agents, including HKMm, the reduction in mycobacterial loads was not as dramatic as with HKLm (Fig. 1B). Importantly, HKLm priming was the most efficient way to induce bacterial clearance. The frequency of clearance increased by 4.8-fold compared to PBS-primed controls. For paclitaxel, priming led to an increase in mycobacterial loads. With the right dosing, paclitaxel can induce proinflammatory responses (Bracci et al., 2014) but can also be toxic to immune cells (Tang et al., 2017), which might explain the increase in mycobacterial load by paclitaxel. Our results indicate that early immune priming affects mycobacterial loads at 7 wpi and that HKLm is the most promising priming agent with our experimental setup.

To ensure that the reduction in mycobacterial loads in adult fish was not due to direct bactericidal effects of HKLm priming, we incubated *M. marinum* in 7H9 medium together with different concentrations of HKLm (Fig. S1). Based on *in vitro* culturing, HKLm does not kill *M. marinum*, indicating that the lowered mycobacterial burdens in zebrafish induced by HKLm priming is not due to direct killing of the mycobacteria by HKLm, but is mediated through effects on the host immune system.

To characterize the duration of the effect after HKLm priming, we injected fish at either 1 or 7* *days prior to infection with *M. marinum*. The mycobacterial loads were determined already at 4 wpi because, at this stage of the infection, bacterial counts have generally reached a steady state and they are easily measurable. Priming 1* *day prior to infection was most efficient, increasing the frequency of a clearing response by 3.7-fold compared to the control group (7 days: 2.3-fold) in this preliminary experiment (Fig. 1C). Based on these results, we continued with HKLm priming at 1 day before *M. marinum* infection.

### Priming with HKLm increases the frequency of clearance of mycobacterial infection in adult zebrafish

**Fig. 2.**
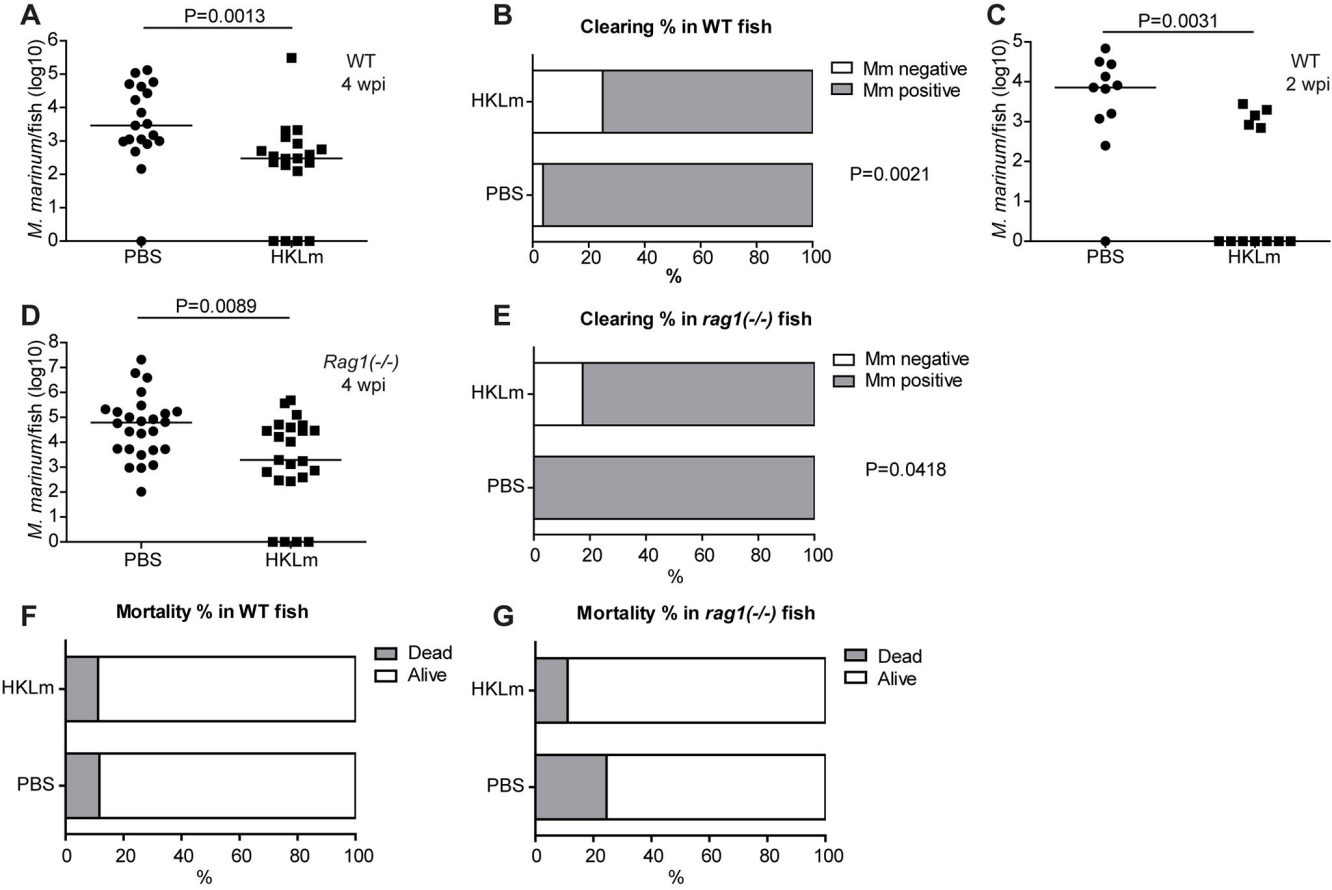
**Priming with HKLm significantly reduces mycobacterial loads in adult zebrafish via innate responses.** (A) Priming of adult zebrafish with 0.5×10^7^ cfu of HKLm 1 day prior to *M. marinum* infection (27±2 cfu) led to a significant decrease in mycobacterial loads compared to control injection of sterile 1× PBS. The graph shows one representative experiment. Samples were collected at 4 wpi (PBS: *n*=19, HKLm: *n*=19). (B) Priming with HKLm 1 day prior to *M. marinum* infection leads to sterilization of *M. marinum* in 25% of the wild-type (WT) zebrafish. Clearance percentage in the WT PBS control group was 3.7%. The data were collected from four independent experiments. *M. marinum* infection doses in the independent experiments were 27±2 cfu, 26±13 cfu, 75±13 cfu and 26±8 cfu. PBS: *n*=54, HKLm: *n*=56. (C) Priming of adult zebrafish with HKLm leads to a significant decrease in mycobacterial loads compared to PBS controls already at 2 wpi. Infection dose: 48±8 cfu. PBS: *n*=11, HKLm: *n*=12. (D) HKLm priming 1 day prior to *M. marinum* infection significantly reduced mycobacterial loads in *rag1*^−/−^ mutant fish compared to the PBS control group at 4 wpi, indicating a role for innate immune responses. The graph contains a combined result from two separate experiments. *M. marinum* infection doses were 48±8 cfu and 27±2 cfu. (E) Priming with HKLm 1 day prior to *M. marinum* infection leads to sterilization of *M. marinum* in 17% of the *rag1*^−/−^ mutant fish. Clearance percentage in the PBS control group was 0%. Data are pooled from two independent experiments. PBS: *n*=26, HKLm: *n*=23. (F,G) HKLm priming did not affect cumulative mortality in WT adult fish (F; PBS: *n*=152, HKLm: *n*=170), but caused a trend of reduced mortality in HKLm-injected *rag1*^−/−^ mutant fish (PBS: *n*=53, HKLm: *n*=55). *P*-values in A, C and D were calculated with a two-tailed non-parametric Mann–Whitney test with GraphPad Prism. Medians for the individual experiments are shown in the figures. The *P*-values in B, E, F and G were calculated with Fisher's test using GraphPad (QuickCalcs) online software.

To verify our finding from the preliminary priming experiments, zebrafish were primed with an injection of HKLm (Fig. 2A) 1 day prior to *M. marinum* infection, and PBS was used as a negative control. Compared to control treatments, priming with HKLm 1 day before infection consistently led to a significant decrease (14.1-fold decrease, on average) in mycobacterial loads in adult zebrafish at 4 wpi (Fig. 2A shows a representative result of four separate experiments).

From our previous study, we know that, without priming, approximately 10% of *M. marinum-*infected zebrafish are able to naturally clear the mycobacterial infection (Hammarén et al., 2014). Combining the results from four different experiments shows that priming with HKLm 1 day prior to *M. marinum* infection increases the frequency of sterilizing response by 6.8-fold in the wild-type fish at 4 wpi [PBS 3.7% (*n*=54) vs HKLm 25% (*n*=56), *P*=0.0021] (Fig. 2B). However, individuals that have been unable to clear the infection also benefit from HKLm priming in terms of lowered bacterial loads (Fig. S2). In a wild-type population with sustained infection, HKLm reduced the median bacterial load by 11.1-fold (*P*<0.0001).

### Innate immune mechanisms mediate HKLm-induced protection against mycobacterial infection

Our results show a beneficial effect of HKLm priming on mycobacterial loads and clearance at 7 wpi (Fig. 1B) and 4 wpi (Fig. 2A), as well as at 2 wpi (Fig. 2C), all of which are late enough stages for both innate and adaptive responses to be active, although it is known that virulent mycobacteria cause a delay in the activation of T-cell responses (Gallegos et al., 2008). To test whether HKLm priming has the same reducing effect on mycobacterial loads in the absence of an adaptive immune response, we used *rag1^−/−^* *^^*mutant fish. Fish lacking *rag1* do not undergo V(D)J recombination, which is essential for the production of the full variety of T- and B-cell receptors. The *rag1^−/−^* *^^*mutants therefore rely solely on their innate immune mechanisms for protection against infections (Wienholds et al., 2002).

When *rag1^−/−^* *^^*mutants were primed with HKLm 1 day before *M. marinum* infection and bacterial loads were measured with qPCR at 4 wpi, HKLm priming caused a similar reduction in bacterial loads in *rag1^−/−^* *^^*mutants (30-fold, *P*=0.0089) as in wild-type fish (9.7-fold, *P*=0.0013) (Fig. 2D,A). Also in *rag1^−/−^*  mutants, priming with HKLm increased the frequency of sterilizing response from 0% (*n*=26) to 17% (*n*=23) (*P*=0.0418) (Fig. 2E). Looking at the fish with a sustained infection (unable to clear the infection), HKLm priming reduced the bacterial load in *rag1* mutants by 7.2-fold (Fig. S2B, not statistically significant). HKLm priming did not affect the cumulative mortality of low-dose-infected wild-type adult fish (PBS: 11.7%; HKLm: 11.2%) but caused a trend of reduced mortality in *rag1^−/−^* **mutant fish (PBS: 24.5%; HKLm: 11.1%) (Fig. 2F,G). Together, these results imply an important role for innate immunity in the formation of protective HKLm-induced immune responses against mycobacterial infection. Importantly, they show that an optimal response induced by HKLm priming significantly increases the frequency of clearance of mycobacterial infection also in the absence of adaptive immunity.

### Protective effects of HKLm priming are not seen in the larval *M. marinum-*infection model

As our results showed that the protective effects of HKLm priming can be mediated through innate immune responses alone, we next used zebrafish larvae to test the effect of HKLm priming on *M. marinum* infection. During the first weeks of development, zebrafish lack adaptive immune responses and rely solely on innate immune responses, making it possible to study mechanisms of mycobacterial infection that arise from innate immunity. To this end, zebrafish larvae were primed with HKLm to induce a protective immune response against *M. marinum* infection. Zebrafish were primed by intravenous injection either at 1 day post-fertilization (dpf) (Fig. S3A) or at 2 dpf (Fig. S3B). *M. marinum* infection was carried out intravenously at 2 dpf. Within this experimental setting, mycobacterial loads were not reduced in zebrafish larvae after priming with HKLm (Fig. S3), possibly due to the immaturity of immune responses in the young larvae or due to the relatively lower HKLm dose that could be delivered into the larvae*.* As we were unable to see any protective effect in the zebrafish larvae, we continued using adult zebrafish for subsequent experiments.

### Protective immunity against *M. marinum* infection is mediated by a protein and/or nucleic acid component of HKLm

**Fig. 3.**
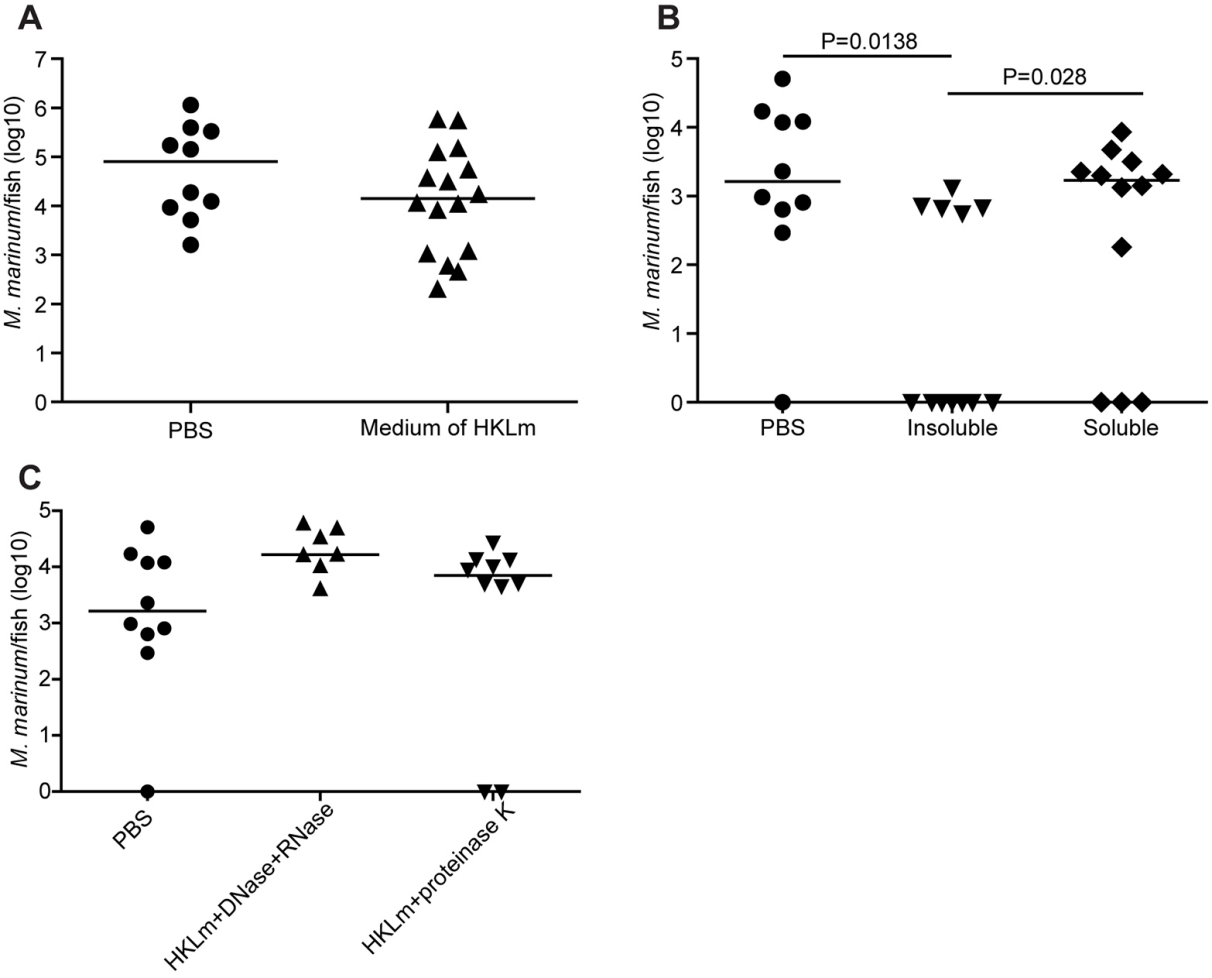
**Protective immunity against *M. marinum* is mediated by a protein and/or nucleic acid component of HKLm.** (A-C) Zebrafish were primed with different components of HKLm and *L. monocytogenes* 1* *day prior to *M. marinum* infection and mycobacterial loads were determined with *M. marinum*-specific qPCR at 4 wpi. (A) Protective immunity is not mediated by a secreted component in the *L. monocytogenes* growth medium. Infection dose: 26±6 cfu; PBS: *n*=10, medium: *n*=16. (B) Protective immunity is mediated by a component that was found in the insoluble phase. Infection dose: 33±11 cfu; PBS: *n*=10, insoluble: *n*=12, soluble: *n*=12. (C) Protective effect of HKLm priming was lost when HKLm was treated with DNase and RNase, or proteinase K. Infection dose: 33±11 cfu; PBS: *n*=10, DNase and RNase: *n*=7, proteinase K: *n*=8. *P*-values for all experiments were calculated with a two-tailed non-parametric Mann–Whitney test with GraphPad Prism and corrected with the Bonferroni's method. Medians for each experiment are shown.

Based on the results of the preliminary experiment with different priming agents, HKLm seemed to contain a specific component responsible for the induction of sterilizing immunity. To characterize the protective component(s), we carried out a set of experiments in which zebrafish were primed with different preparations of HKLm 1* *day prior to *M. marinum* infection and bacterial growth was analyzed at 4 wpi. Priming with spent *L. monocytogenes* medium did not cause a significant reduction in the mycobacterial loads of the fish, indicating that a secreted factor was not the causative agent behind the activation of antimycobacterial immune responses (Fig. 3A). We also found that, after autoclavation of *L. monocytogenes*, priming with the insoluble material provided a protective effect against *M. marinum* infection, whereas the soluble material was not protective (Fig. 3B). However, we were able to show that treatment of the HKLm extract with either proteinase K or a cocktail of RNase and DNase abolished the protective effect (Fig. 3C). These results indicate that the protective component is stable, resistant to high temperature and pressure, non-secreted, insoluble, and likely consists of both a protein and nucleic acid component.

### Therapeutic potential of HKLm treatment

**Fig. 4.**
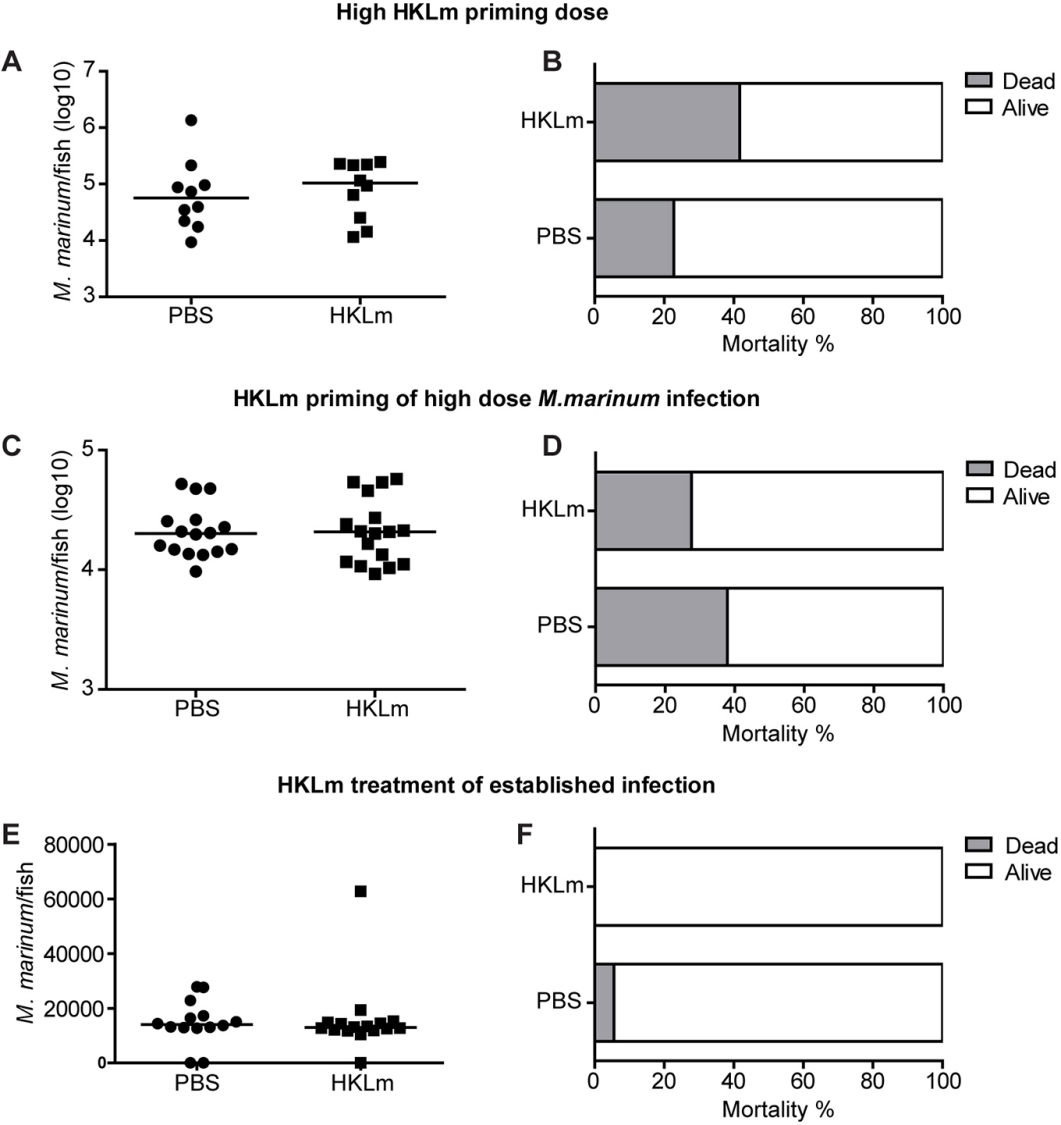
**HKLm treatment does not protect against high-dose or established *M. marinum* infection.** (A,B) Protective effect of HKLm priming is lost with high-dose priming. Fish were injected with a high dose of HKLm (15.6×10^7^ cfu, 30-fold compared to previous dose) 1* *day prior to *M. marinum* infection (34±11 cfu). Priming with a high dose of HKLm did not reduce mycobacterial numbers (A) and led to an increase in the mortality of the fish at 4* *wpi (B). PBS: *n*=10, HKLm: *n*=10. (C,D) HKLm priming does not protect from high-dose *M. marinum* infection. Fish were primed with HKLm 1* *day prior to high-dose *M. marinum* infection (4883±919 cfu). No effect was observed on bacterial loads (C) or cumulative end-point mortality (D). PBS: *n*=16, HKLm: *n*=17. (E,F) HKLm does not protect against an established *M. marinum* infection. Fish were injected with HKLm 2 weeks after an *M. marinum* infection (22±6 cfu). No effect on mycobacterial loads (E) or cumulative end-point mortality (F) was observed. PBS: *n*=14, HKLm: *n*=16. *P*-values for bacterial loads were calculated with a two-tailed non-parametric Mann–Whitney test with GraphPad Prism (A,C,E). Medians for the experiments are shown.

To further characterize the features of HKLm treatment, a 30-fold higher HKLm dose was injected 1* *day prior to low-dose *M. marinum* infection. With such a high dose, HKLm lost its beneficial effects (Fig. 4A) and there was a trend of increased mortality of the fish by 4 wpi (PBS: 22.7%; HKLm: 41.7%) (Fig. 4B). According to these results, it was concluded that, by increasing the HKLm dose, it is not possible to further boost the protective effect but rather *vice versa*. The optimal dosage thus plays a crucial role in inducing a protective response.

We then tested whether priming with HKLm 1* *day prior to infection could protect fish against a high-dose infection challenge with 4883±919 colony-forming units (cfu) of *M. marinum*. In the context of a high-dose infection, HKLm did not induce significant difference in the mycobacterial loads, clearance or cumulative end-point mortality at 4 wpi (Fig. 4C,D).

We next wanted to test whether HKLm can induce protective immune responses if the mycobacterial infection is already established. We have previously shown in adult zebrafish that, at 2 wpi, *M. marinum* infection is well established and granulomas have already started to form (Parikka et al., 2012). We infected adult zebrafish with a low dose of *M. marinum*, injected HKLm at 2 wpi and measured mycobacterial loads, as well as determined the cumulative mortality at 4 wpi. Based on our results, at a time point in which the mycobacteria have already multiplied substantially and started forming granulomas, HKLm injection was not able to lower the bacterial loads, increase bacterial clearance or affect cumulative mortality (Fig. 4E,F).

### Priming adult zebrafish with HKLm induces *mpeg1*, *tnfα* and *nos2* expression, and downregulates *sod2*, at the early phase of mycobacterial infection

**Fig. 5.**
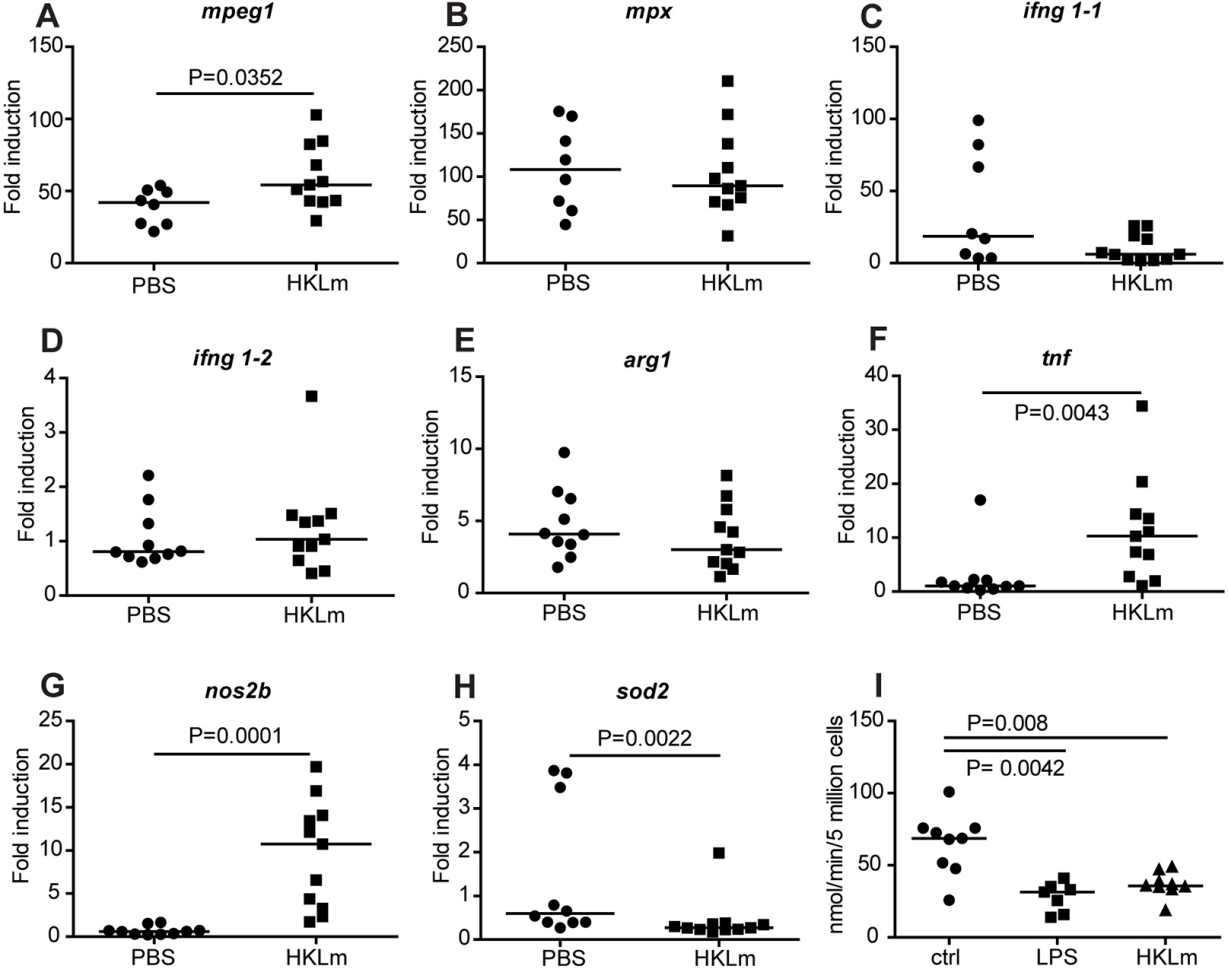
**HKLm priming induces *mpeg*, *tnfα* and *nos2b* expression, downregulates *sod2* expression in adult zebrafish and leads to decreased oxygen consumption *in vitro*.** (A-H) The expression levels of *mpeg* (A), *mpx* (B), *ifnγ1-1* (C), *ifnγ1-2* (D), *arg1* (E), *tnfα* (F), *nos2b* (G) and *sod2* (H) were measured with qPCR from wild-type fish primed with HKLm or sterile PBS buffer as a control. At 1 day after the priming, the fish were infected with a low dose (67±16 cfu) of *M. marinum*. Samples for qPCR analysis were collected at 1 dpi. The results were normalized to uninfected wild-type baseline control. PBS: *n*=10, HKLm: *n*=11. (I) HKLm priming leads to metabolic changes *in vitro.* RAW264.7 cells were primed with LPS or HKLm and oxygen consumption was measured 19-24 h after priming. HKLm priming leads to a 1.8-fold decrease (*P*=0.008) in oxygen consumption compared to control. LPS was used as a positive control (2.3-fold decrease, *P*=0.0042). PBS: *n*=9, LPS: *n*=7, HKLm: *n*=9. *P*-values for all experiments were calculated with a two-tailed non-parametric Mann–Whitney test with GraphPad Prism. Medians for each experiment are shown. Bonferroni correction was used in C.

We were interested in further characterizing the nature of the immune response induced by HKLm priming in adult zebrafish. We hypothesized that the protective effect could be mediated through enhanced killing of mycobacteria at the early phase of the infection due to changes in the numbers or activity of innate immune cells. To study the details of HKLm-induced immune activation by qPCR, the fish were primed with HKLm or PBS, infected with a low dose of *M. marinum* 1* *day after priming and the total RNA was extracted from zebrafish organs collected 1* *day post-infection (dpi). First, the possible effects of HKLm treatment on the number of macrophages and neutrophils were assessed by measuring the expression of *macrophage expressed gene 1* (*mpeg1*) (Ellett et al., 2011) and *myeloid-specific peroxidase* (*mpx*) (Lieschke et al., 2001), respectively. In the HKLm group, *mpeg1* expression was significantly higher than in the control group (Fig. 5A; HKLm: 59.9-fold; PBS: 39.3-fold; *P*=0.0352), suggesting that the number of macrophages was increased due to HKLm priming. The expression of *mpx* was not affected by HKLm (Fig. 5B).

To assess the HKLm-induced changes at the level of immune cell activation, we measured the expression of a selection of markers related to the effective antimycobacterial functions of innate immune cells 1 day after *M. marinum* infection. Based on literature on the mechanisms limiting intracellular mycobacterial growth, the genes chosen for analysis were *tnfα* (Cobat et al., 2015; Roca and Ramakrishnan, 2013), *ifnγ* (Flynn et al., 1993) and *nos2b* (Nicholson et al., 1996; Thoma-Uszynski et al., 2001). Expression of *ifnγ* was not differentially induced between PBS- and HKLm-treated groups (Fig. 5C,D). The median expression levels of *tnfα* (Fig. 5F; HKLm 10.3-fold vs PBS 1.1-fold, *P*=0.0043) and *nos2b* (Fig. 5G; HKLm 10.7-fold vs 0.6-fold PBS, *P*=0.0001) were significantly increased in the HKLm-primed group compared to the PBS control group. We also analyzed the expression levels of *sod2*, which encodes a mitochondrial protein that converts the byproducts of oxidative phosphorylation to hydrogen peroxide and oxygen, leading to neutralization of mitochondrial reactive oxygen species (ROS) (Pias et al., 2003) and *arg1*, which is an alternative macrophage activation marker (Gordon and Martinez, 2010). In the HKLm group, *sod2* expression was significantly more downregulated as compared to the PBS group, indicating increased levels of ROS due to HKLm priming (PBS 0.59-fold vs HKLm 0.27-fold, *P*=0.0022) (Fig. 5H). *Arg1*, however, was not induced in HKLm-primed fish, suggesting that alternative activation does not have an impact on the early mycobacterial elimination (Fig. 5E). Together, these results suggest that HKLm induces M1-type classical macrophage activation leading to enhanced intracellular killing at the early stages of a mycobacterial infection.

### Priming of macrophages with HKLm leads to decreased oxygen consumption *in vitro*

Classically activated M1 macrophages have been shown to change their metabolism upon immune activation (Cheng et al., 2014). To test whether this is also the case with HKLm priming, we set up a RAW264.7 cell culture and primed them either with LPS (50 ng/µl) or HKLm (MOI: 530). At 19-24 h later, oxygen consumed by these cells was measured using a Clark electrode. We used LPS as a positive control and showed that both treatments lower the oxygen consumption significantly (Fig. 5I). Pooled result from three independent experiments concluded that HKLm- or LPS-primed mouse macrophages consume, respectively, 1.8-times (*P*=0.008) and 2.3-times (*P*=0.0042) less oxygen compared to untreated cells, *in vitro* (Fig. 5I). This result implies that HKLm-treated macrophages exhibit metabolic changes indicative of diminished oxygen similar to those observed during classical macrophage activation.

## DISCUSSION

Despite substantial progress in the field of medicine, TB still kills millions of people every year and has been declared a global public health crisis (World Health Organization, 2016; http://www.who.int/tb/publications/2016/en/). The advances in the battle against TB have been hindered by the complex nature of the disease and the limitations of tuberculosis animal models. TB manifests itself in a wide spectrum of disease, with most affected individuals being unable to eradicate the causative bacteria (Mtb), leading to the development of latent TB infection, which has a lifetime risk of reactivation in 5-10% of cases. The World Health Organization has estimated that 2-3 billion people are latently infected with Mtb (World Health Organization, 2016), creating a huge pool of individuals with a potential to develop an active, transmissive disease. As current vaccination and antibiotic schemes have proven insufficient for controlling the global TB epidemic, host-directed therapies inducing protective immune responses are emerging as a novel strategy to treat TB (Tobin, 2015). Efficient host-directed therapies used either alone or in combination with antibiotics are an approach that could potentially lead to sterilization of TB.

Although genome-wide association studies carried out in humans have given important clues on the nature of protective immune responses against TB (Azad et al., 2012; Wilkinson et al., 2000; Cobat et al., 2009, 2015), animal models are essential for the execution of more mechanistic studies. The mouse, rabbit and macaque have been widely used to study TB (Myllymäki et al., 2015). To the best of our knowledge, spontaneous or induced sterilizing immunity has not been observed at the organismal level in mammalian animal models of TB. Clearance of cultivable mycobacteria occurs in the rabbit model (Subbian et al., 2012). However, standard bacterial culturing methods only detect the actively replicating mycobacterial populations, but not dormant bacteria (Chao and Rubin, 2010), and, in the rabbit, the clearance of cultivable mycobacteria indicates the establishment of a truly latent disease instead of sterilization (Subbian et al., 2012). The zebrafish has recently become a well-accepted genetically tractable vertebrate model for human TB pathogenesis to complement the more traditional mammalian models (Myllymäki et al., 2015). In our previous study, using a qPCR-based method, we were able to see spontaneous clearance of mycobacterial infection in the zebrafish-*M. marinum* infection model in approximately 10% of the fish at 4 wpi (Hammarén et al., 2014). As we observed no clearance at 2 wpi (Hammarén et al., 2014), spontaneous early clearance (likely induced by innate mechanisms) in our wild-type zebrafish population is a rare event. In this current study, we were able to increase the frequency of sterilizing mycobacterial infection by priming the innate immune response prior to *M. marinum* infection.

Our hypothesis was that priming or stimulation of the immune response in the adult zebrafish before *M. marinum* infection could lead to a sterilizing immune response instead of a lethal primary active disease or a latent infection that is prone to reactivation later in life (Parikka et al., 2012). By using an array of different priming agents, we wanted to study whether we could create a climate that would allow the immune response to circumvent their efficient virulence strategies and even eradicate the mycobacteria. Priming approaches have been successful in inducing protective responses against various other bacterial infections in the fruit fly, *Drosophila melanogaster*, which relies solely on innate immunity (Pham et al., 2007). Indeed, our results showed that sterilizing immunity can be induced in the *M. marinum* zebrafish model. In our hands, priming with HKLm induces a sterilizing response in 25% of *M. marinum*-infected fish at 4 wpi, whereas the percentage of spontaneous clearance was 3.7% in the PBS-primed control group.

The *M. marinum*-specific qPCR used to quantify bacterial loads is a sensitive method, with a detection limit of approximately 100 bacterial genomes in the entire fish (Parikka et al., 2012; Hammarén et al., 2014). The major advantage of a qPCR-based method over culturing methods is that qPCR detects all bacterial genomes irrespective of metabolic state so that dormant bacteria are also detected. An advantage of the zebrafish is that, due to its small size, all infection target organs can be collected for determination of bacterial load, which is not practically feasible in larger animals. Based on the qPCR results, we are able to say that 25% of the wild-type fish were able to clear the infection, indicating that HKLm priming indeed increases the frequency of sterilizing immune response. This model provides tools for elucidating the detailed mechanisms behind sterilizing immunity against TB.

To study whether the protective effects of HKLm priming require a functional adaptive immune system, we used *rag1* mutant zebrafish. Although *rag1* mutant zebrafish are known to be hypersusceptible to *M. marinum* infection due to a failure in the development of functional T and B cells (Swaim et al., 2006; Parikka et al., 2012), their bacterial loads were significantly lowered and even cleared by HKLm priming, suggesting that the protective response can be mediated by innate immunity alone. The role of innate immunity is also supported by the significant HKLm-induced reduction in bacterial loads as early as 2 wpi in wild-type fish, by which time adaptive responses are only starting to arise in mycobacterial infections (Andersen and Woodworth, 2014). Despite the clear involvement of innate responses in HKLm-induced protective responses in adult fish, the results could not be reproduced in young zebrafish larvae that have only innate immunity, probably due to an immature immune response and technical difficulties to deliver high enough doses of HKLm by microinjection methods. As adaptive responses are centrally involved in the pathogenesis of mycobacterial infections, using the adult zebrafish will likely better model the effects of immunomodulatory treatments on the immune response as a whole. On top of the protective effects of HKLm mediated through innate immunity, an additional level of protection was observed in the presence of a functional adaptive immune system in zebrafish. However, the observation that activation of innate immunity alone by immune priming with HKLm in some cases was sufficient for induction of a protective or sterilizing immunity opens new avenues for host-directed therapies or preventive strategies even in the absence of a fully functional adaptive immune system, such as in patients with an HIV co-infection.

At the moment, there is no immunotherapy that could cure an ongoing mycobacterial infection by simply boosting the immune system. Therefore, we wanted to study whether HKLm could induce a protective response during an established mycobacterial infection. A single dose of HKLm was injected 2 weeks after *M. marinum* infection, when early granulomas have started to form and are visible in various organs (Parikka et al., 2012). However, this single injection of HKLm did not decrease mycobacterial loads at 4 wpi (Fig. 4E,F). By this time point, *M. marinum* has already had plenty of time to exert its early virulence strategies leading to effective avoidance of the pro-inflammatory host immune response (Elks et al., 2014; Queval et al., 2016; Bhat et al., 2017; Cambier et al., 2014). Having gained a foothold within its host, mycobacteria are not as prone to the effects caused by a single therapeutic injection of HKLm as they are when entering a primed host. Adult zebrafish have also been used to model active fulminant TB by infecting individuals with a high *M. marinum* dose (Parikka et al., 2012). Individuals infected with a high *M. marinum* dose did not benefit from HKLm priming. The high infection dose of a few thousand mycobacteria leads to a disease state in which the capacity of the immune system rapidly becomes saturated, allowing the bacteria to grow almost logarithmically (Parikka et al., 2012) possibly due to the limited number of macrophages (Pagán et al., 2015) compared to the low infection dose; a situation too challenging to overcome even in the presence of HKLm priming. However, in our preliminary experiments, we saw that, in addition to priming 1* *day prior to infection, protective effects were still visible when HKLm priming was delivered 1 week prior to *M. marinum* infection (Fig. 1C), suggesting that this type of protective response could be considered in designing new preventive strategies.

The protective effects of HKLm treatment delivered prior to infection could be mediated through the induction of trained immunity. It is known that innate immune cells can mediate an enhanced immune response upon reinfection with the same pathogen (Quintin et al., 2012). The innate immune system can also cross-react with a new pathogen according to previous stimuli (Kleinnijenhuis et al., 2014). It has been reported that some vaccines can produce durable cross-protection that cannot be explained by adaptive responses (Aaby et al., 2014). This type of nonspecific innate memory is referred to as trained immunity. Mechanisms of trained immunity have also been shown to be responsible for BCG-induced by-stander protection against *Candida albicans* in the mouse (Van't Wout et al., 1992). Innate memory is mediated through reversible epigenetic changes rather than irreversible genetic recombination seen during the formation of classical immunological memory in adaptive immune cells and can last for weeks to months (reviewed by Netea et al., 2016). The effects of trained immunity in the context of susceptibility to TB is an interesting area of research and can yield new approaches in the development of preventive strategies based on innate immunity.

It has been reported that the number of macrophages is critical for the disease outcome and that macrophage deficiency is connected to accelerated progression of mycobacterial infection (Pagán et al., 2015). To assess the effect of HKLm priming on the number of macrophages and neutrophils, we measured the expression of the commonly used markers *mpeg1* (Ellett et al., 2011) and *mpx* (Lieschke et al., 2001), respectively. Based on the expression of these markers, the number of macrophages was significantly higher in HKLm-primed fish (*P*=0.0352), whereas the amount of neutrophils remained unchanged (Fig. 5A,B). This change seen in macrophages is potentially mediating the protective response against mycobacteria.

To decipher the type of protective immune response induced by HKLm priming, we measured an array of genes related to innate immune activation in the organs of *M. marinum-*infected zebrafish at 1 dpi. In line with *in vitro* studies on *Listeria* (Barbuddhe et al., 1998; Mirkovitch et al., 2006), *in vivo* priming with HKLm caused a significant increase in *nos2b* and *tnfα* with a simultaneous decrease in *sod2* expression. *nos2b* is one of the NO synthases in zebrafish (Lepiller et al., 2009). NO is known to be mycobacteriocidal (Nicholson et al., 1996) but, as pathogenic mycobacteria have developed evasion strategies to inhibit the production of NO (Elks et al., 2014; Queval et al., 2016; Bhat et al., 2017), the NO levels naturally induced in Mtb-infected macrophages seem to be insufficient for lysing mycobacteria (Jung et al., 2013). Thus, the additional production of Nos caused by HKLm prior to infection likely potentiates the intracellular killing mechanisms. The beneficial effects of increased NO in neutrophils (Elks et al., 2013) as well as in macrophages (Cambier et al., 2014) during early *M. marinum* infection have previously been demonstrated in zebrafish larvae. Cambier and colleagues showed that virulent mycobacteria avoid NO-mediated intracellular killing during the early phase of infection by hiding their TLR ligands under a phthiocerol dimycoceroserate coat (Cambier et al., 2014). Co-infecting zebrafish larvae with *M. marinum* and *Staphylococcus aureus* or *Pseudomonas aeruginosa* leads to attenuation of mycobacterial infection (Cambier et al., 2014). Also, co-injection of live *M. marinum* with heat-killed *M. marinum* or with a mutant with exposed TLR ligands caused similar attenuation (Cambier et al., 2014). It is likely that at least part of the protective effects caused by HKLm in the adult zebrafish are mediated through TLR ligands. The component analysis of HKLm suggested that nucleotides were important for HKLm-mediated protection against *M. marinum*. TLR9, which recognizes double-stranded DNA and leads to the induction of Nos2 (Ito et al., 2005), is a receptor potentially responsible for the protection. However, detailed analysis of the signaling pathways activated by HKLm treatment was beyond the scope of this study.

Tnfα is also known to mediate intracellular killing of mycobacteria by macrophages (Roca and Ramakrishnan, 2013) and optimal levels of this cytokine have been proposed to lead to early clearance of TB in humans (Cobat et al., 2015). Studies in zebrafish larvae have shown that high Tnfα levels alongside high ROS levels within macrophages, during the early days of mycobacterial infection, is bactericidal (Roca and Ramakrishnan, 2013). Sod2 is an enzyme that acts through neutralization of mitochondrial ROS (Pias et al., 2003). Its downregulation by HKLm should thus cause an increase in mitochondrial ROS, the high levels of which have been shown to enhance intracellular killing mechanisms within macrophages (reviewed in Hall et al., 2014). Recently, in a human population study, a genetic variant leading to reduced activity of *Sod2* was found to be associated with increased resistance to leprosy, a disease caused by *Mycobacterium leprae* (Ramos et al., 2016). Therefore, a likely mechanism of clearing the mycobacterial infection by HKLm priming in the adult zebrafish model is mediated through increased Tnfα and decreased Sod2 production that together lead to higher, mycobacteriocidal, levels of ROS within macrophages.

Roca and Ramakrishnan also showed that, when the expression of Tnfα is endogenously high, continuously high ROS levels after the first days of infection lead to excessive inflammation, necrosis and exacerbation of the disease (Roca and Ramakrishnan, 2013). This probably also explains the increased mortality with the higher HKLm dose. With a single small dose of HKLm used in our study, the effects of the treatment were undoubtedly positive, but it must be kept in mind that excessive or prolonged induction of Tnfα and ROS can also have detrimental effects. The dosage of treatment as well as the genotype of the host, affecting the baseline production of inflammatory cytokines, will also need to be carefully considered in the development of host-directed immunomodulatory treatments.

Based on the gene expression data, the changes in the innate immunity induced by HKLm in the adult zebrafish seem to be mediated through an increased number and activation of M1-type macrophages. Recent research on the metabolism of different innate immune cells has shown that M1 macrophages have decreased oxygen consumption, and increased glycolysis and lactate production (Cheng et al., 2014). In our experiments, the oxygen consumption of mouse macrophages was significantly decreased by HKLm priming (Fig. 5I), providing a further piece of evidence of M1 macrophages playing a central role in HKLm-mediated protection against mycobacterial infection. The result also implies that the types of activation caused by HKLm in the zebrafish are similar to those induced in mammalian macrophages.

Overall, we show that protective and even sterilizing immune responses can be induced in the zebrafish model for TB by priming with HKLm*.* The response is induced even in the absence of adaptive immunity and is accompanied by the increase in the number of macrophages, the induction of *tnfα* and *nos2b*, and the downregulation of *sod2*, likely leading to increased production of radical nitrogen and oxygen species and enhanced intracellular killing of mycobacteria. Based on our results, it seems that the type of activation induced by HKLm treatment is only effective when delivered at an early enough time point prior to exposure to pathogenic mycobacteria. The model provides a platform in which both innate and adaptive mechanisms leading to sterilization of mycobacterial infection can be reliably studied. Such knowledge will contribute to the development of new vaccination strategies as well as host-directed therapies aimed at prevention of transmission and sterilizing treatment of TB disease.

## MATERIALS AND METHODS

### Zebrafish lines and housing

Adult 5- to 10-month-old male and female AB wild-type zebrafish (*Danio rerio*) and *rag1^−/−^*  (hu1999) mutant zebrafish (from Zebrafish International Resource Center, University of Oregon, OR, USA) were used in the experiments. The fish were housed in flow-through water-circulation systems with a 14 h/10 h light/dark cycle.

### Ethics statement

All experiments were conducted according to the Finnish Act on Animal Experimentation (62/2006) and the Act on the Protection of Animals Used for Scientific or Educational Purposes (497/2013). ELLA (Eläinkoelautakunta; the National Animal Experiment Board in Finland under the Regional State Administrative Agency for Southern Finland) approved the Tampere zebrafish facility and the animal experiments carried out in this project under the licenses ESAVI/6407/04.10.03/2012, ESAVI/8245/04.10.07/2015 and ESAVI/10079/04.10.06/2015.

### Experimental *M. marinum* infections

*Mycobacterium marinum* (ATCC 927) was first pre-cultured on Middlebrook 7H10 plates with OADC enrichment (Fisher Scientific, NH, USA) at 29°C for 1 week. After plate culturing, *M. marinum* was transferred into Middlebrook 7H9 medium with ADC enrichment (Fisher Scientific, NH, USA) with 0.2% Tween-80 (Sigma-Aldrich, MO, USA), cultured for 3-4* *days, diluted 1:10 and cultured for a further 2* *days until OD600 nm reached 0.460-0.650. For adult zebrafish infections, *M. marinum* was first harvested by centrifuging for 3 min at 10,000 ***g*** and was then resuspended and diluted in sterile 1× PBS with 0.3 mg/ml of Phenol Red (Sigma-Aldrich, MO, USA). A total of 5 µl of the suspension (33±19 cfu/fish) was injected i.p. with an Omnican 100 30 G insulin needle (Braun, Melsungen, Germany) under 0.02% 3-aminobenzoic acid ethyl ester (pH 7.0) (Sigma-Aldrich, MO, USA) anesthesia. Infection doses were verified by plating 5 µl of the injection suspension on a 7H10 plate.

For larval infections, the *M. marinum* pTEC15 strain was used. This in-house-made *M. marinum* wasabi-fluorescent strain was made by transforming a pTEC15 plasmid (Addgene plasmid #30174, deposited by Lalita Ramakrishnan; Takaki et al., 2013) into the *M. marinum* ATCC 927 strain by electroporation. For larval infections, the *M. marinum* pTEC15 strain was cultured for 4-5* *days in supplemented 7H9 medium with 75 µg/ml hygromycin (Merck, Darmstadt, Germany), diluted 1:10, cultured for 3* *days until the OD600 nm was 0.407-0.537 and harvested for infection by centrifugation.

### Zebrafish larval infection experiments

To study the effect of HKLm priming in zebrafish larvae, 1 nl of HKLm (240 cfu) or PBS control were injected into the caudal vein at 1 dpf under 0.0045% 1-phenyl-2-thiourea (Sigma-Aldrich, MO, USA) anesthesia. At 2 dpf the larvae were infected with 1 nl of *M. marinum* (39±13 cfu) into the blood circulation valley, transferred to fresh E3 medium and kept at 29°C. At 8 dpi, larvae were collected for DNA extraction with TRI Reagent (Fisher Scientific, NH, USA). DNA extraction was performed according to the manufacturer's instructions, after which *M. marinum*-specific qPCR was used to quantify mycobacteria.

For the quantification of pTEC15 fluorescence, 1-dpf wild-type AB embryos were dechorionated and kept in E3 medium with 0.0045% 1-phenyl-2-thiourea (Sigma-Aldrich, MO, USA) at 29°C to prevent pigmentation. A total of 1 nl of wasabi-fluorescent *M. marinum* suspension (39±16 cfu) with 0.6 mg/ml of Phenol Red (Sigma-Aldrich, MO, USA) and 490 cfu of HKLm were microinjected into the blood circulation valley at 2 dpf with a glass microcapillary. *M. marinum* infection doses were verified by plating the injection doses on a 7H10 agar plate. After infection, the larvae were kept in E3 medium with 0.0045% 1-phenyl-2-thiourea (Sigma-Aldrich, MO, USA) on 24-well plates at 29°C.

At 7 dpi, larvae were anesthetized with 0.02% 3-aminobenzoic acid ethyl ester (pH 7.0) (Sigma-Aldrich, MO, USA). The larvae were embedded on their side in 1% low-melt agarose in E3 medium on black 96-proxiplates (Perkin-Elmer, MA, USA). Extra E3 medium with the anesthetic was added on top of the solidified low-melt agarose to prevent the larvae from drying. The wasabi-fluorescent signal was measured three times using the EnVision plate reader (Perkin-Elmer, MA, USA) scanning program. The scan measurement was carried out on five horizontal and five vertical dots 0.5 mm apart from 6.5 mm height with 100% excitation at 493 nm, 509 nm emission and 500 flashes per point. The fluorescent signals of individual zebrafish larvae were normalized with the average signal from healthy non-infected larvae.

### Preparation of heat-killed bacteria and priming injections

For the preparation of heat-killed bacteria, *L. monocytogenes* (10403S), *E. coli* (ATCC 25922), *S. aureus* (ATCC 29213), *S. typhimurium* (ATCC 14028) and *S. iniae* (ATCC 29178) were inoculated from glycerol stocks or blood agar plates and cultured in brain heart broth (BHB) (Sigma-Aldrich, MO, USA) at 37°C until the OD600 nm reached 0.9-1.0. Bacterial suspensions were plated on LB agar plates to verify bacterial concentrations. To heat-kill bacteria, the bacterial suspensions were autoclaved in BHB at 120°C for 20 min and the sterility was confirmed by plating on LB plates after autoclaving. Injection doses of heat-killed bacteria for adult zebrafish were 0.5×10^7^-1×10^7^ cfu. Injection doses for other priming agents were 13.5 µg/fish for LPS (Sigma-Aldrich, MO, USA), paclitaxel (Sigma-Aldrich, MO, USA) and zymosan (Sigma-Aldrich, MO, USA), and 4.5 µg/fish for muramyl-dipeptide (Sigma-Aldrich, MO, USA). Priming i.p. injections (5 µl) were injected with an Omnican 100 30 G insulin needle (Braun, Melsungen, Germany) under 0.02% 3-aminobenzoic acid ethyl ester (pH 7.0) anesthesia.

### DNase, RNase and proteinase K treatment of HKLm

DNase and RNase treatments were performed for autoclaved *L. monocytogenes* in BHB medium. Heat-killed bacterial suspension was incubated with 10 µg/ml of RNase A (Thermo Fisher Scientific, NH, USA) at 37°C for 18 h. After RNase treatment, the suspension was treated with 83 U/ml DNase I (Thermo Fisher Scientific, NH, USA) according to the manufacturer's instructions. Accordingly, HKLm was treated with 10 µg/ml of proteinase K (Thermo Fisher Scientific, NH, USA) at 37°C for 18 h and inactivated at 70°C for 15 min before injections.

### RNA and DNA extractions from zebrafish samples

For RNA and DNA extractions, adult zebrafish were first euthanized with an overdose of 3-aminobenzoic acid ethyl ester anesthetic and then internal organs were collected from the body cavity. Organs were homogenized in TRI Reagent (Thermo Fisher Scientific, NH, USA) with ceramic beads using the PowerLyzer24 (Mobio, CA, USA) bead beater at 3200 rpm for 3×40 s. Samples were cooled on ice between the cycles. After homogenization, samples were sonicated for 9 min and the RNA and DNA were extracted according to the manufacturer's instructions.

### Gene expression studies and quantifying mycobacterial loads by qPCR

Prior to qPCR analysis, RNA was treated with DNase I (Thermo Fisher Scientific, NH, USA) to remove possible traces of genomic DNA according to the manufacturer's instructions. After DNase treatment, RNA was reverse transcribed into cDNA with a Reverse Transcription kit (Fluidigm, CA, USA) according to the manufacturer's instructions. Gene expression was measured by using SsoFast EvaGreen Supermix with Low ROX qPCR kit (Bio-Rad, CA, USA) with the CFX96 qPCR system (Bio-Rad, CA, USA). Zebrafish genes were normalized with expressed repetitive element *loopern4* (Vanhauwaert et al., 2014) and compared with the average induction of pooled baseline sample of healthy non-infected zebrafish. Results were analyzed using the ΔCt method and are shown as fold induction.

Mycobacterial loads were measured with the SensiFAST SYBR No-ROX qPCR kit (Bioline, London, UK) from genomic DNA according to the manufacturer's instructions. Each bacterial quantification qPCR run included standard curve of known amounts of *M. marinum* DNA. Primer sequences and gene association numbers are shown in Table 1.

**Table 1.**
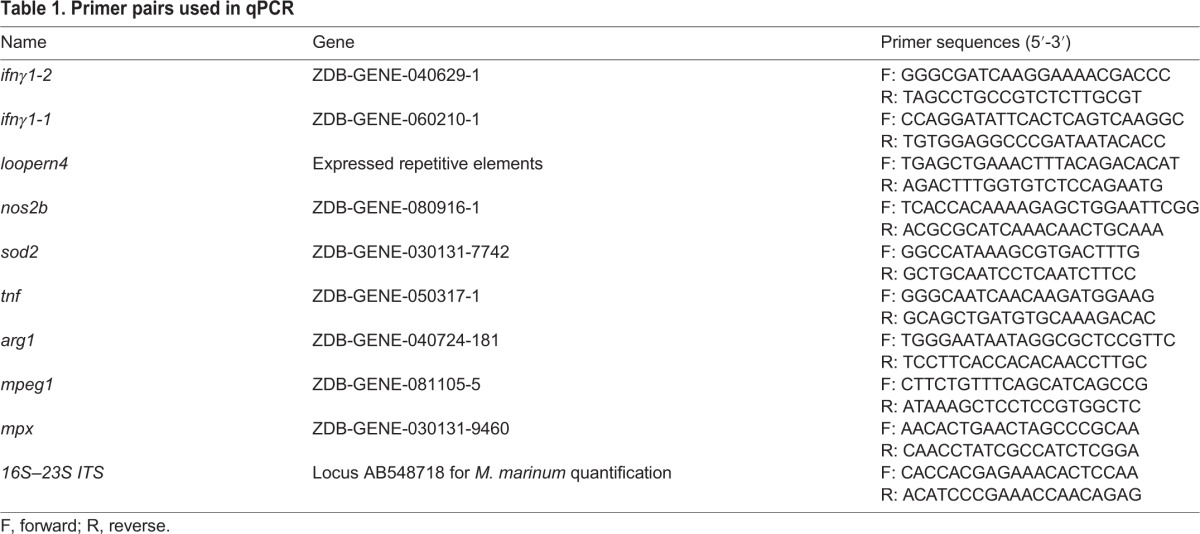
**Primer pairs used in qPCR**

### Measurement of oxygen consumption in HKLm-primed RAW264.7 cells

To measure oxygen consumption, RAW264.7 cells (ATCC TIB-71) were cultured in Dulbecco's modified Eagle's medium with 4.5 g/l D-glucose and L-glutamine (Gibco, Thermo Fisher Scientific, NH, USA) supplemented with 10% of heat-inactivated fetal bovine serum (Gibco, Thermo Fisher Scientific, NH, USA) and 100 U/ml penicillin-streptomycin (Thermo Fisher Scientific, NH, USA) at 37°C with 5% CO_2_. The cells were primed either with 50 ng/ml of LPS or HKLm to correspond to an MOI of 530. At 19-24* *h later the media was changed to fresh media including priming agents. A minimum of 30 min later the oxygen consumption from 5 million cells was measured at 37°C in their culture media using a Clark electrode (Hansatech, UK). Mitochondrial respiration was measured as the total minus the background oxygen consumption; the latter being determined by exposing the cell suspension to 150-270 nM of antimycin A (Sigma-Aldrich, MO, USA), a potent inhibitor of the respiratory chain complex III.

### Statistical analyses

GraphPad Prism software (5.02) was used to carry out statistical analysis. A non-parametric two-tailed Mann–Whitney test was used to compare differences between experimental groups. Bonferroni's post-test was used to correct *P*-values for multiple comparisons. *P*-values smaller than 0.05 were considered as significant. The sample sizes for experimental fish groups were calculated with power and sample size program (version 3.1.2) by using data from our preliminary studies (Dupont and Plummer, 1998).

## Supplementary Material

Supplementary information
