# Supplementary material for: Priming of innate antimycobacterial immunity by heat-killed Listeria monocytogenes induces sterilizing response in the adult zebrafish tuberculosis model
Source: Dis Model Mech. 2018 Jan 1;11(1):dmm031658. doi: 10.1242/dmm.031658 (PMC5818079; doi:10.1242/dmm.031658)
Supplement: Supplementary information [file dmm-11-031658-s1.pdf]

FIRST PERSON

First person – Hanna Luukinen

First Person is a series of interviews with the first authors of a selection of papers published in Disease Models & Mechanisms, helping early-career researchers promote themselves alongside their papers. Hanna Luukinen is first author on 'Priming of innate antimycobacterial immunity by heat-killed *Listeria monocytogenes* induces sterilizing response in the adult zebrafish tuberculosis model', published in DMM. Hanna is a PhD student in the lab of Mataleena Parikka at the University of Tampere, Finland, investigating the mechanisms behind mycobacterial persistence both in the context of evading the host immune responses as well as phenotypic tolerance to antibiotic treatment.

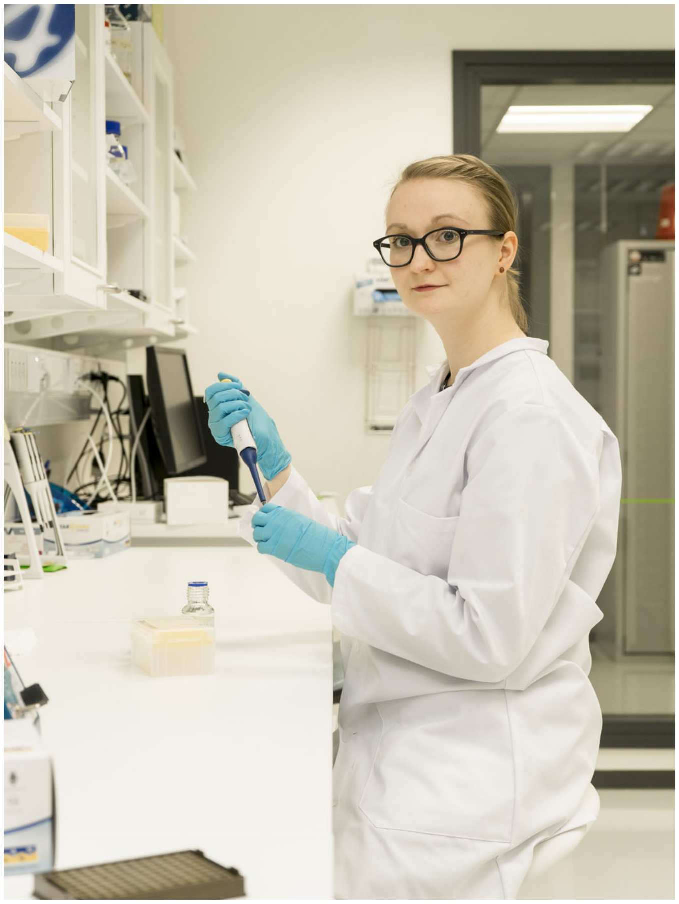

Hanna Luukinen

How would you explain the main findings of your paper to non-scientific family and friends?

When I tell my family and friends that I am doing a PhD on tuberculosis the discussion quite often changes very quickly to what we should eat next. Nourishment is an important topic of course, but if the conversation would continue with my favorite subject, tuberculosis, there are couple of points I would like to make. The causative agent of tuberculosis, mycobacterium, is a master of dampening the immune system and hiding from it, which has made the bacteria hard to eliminate the bacteria. These evasion strategies have also affected vaccine development to the point, that currently, there is no vaccine that is able to protect against the infection. In this study, we found that with a bacterial booster we were able to help individuals to cope with mycobacterial infection and even get rid of this ingenious bacteria. This booster seems to work via the very basic mechanisms of the immune system. It's like margherita pizza – it just works without any additional toppings.

What are the potential implications of these results for your field of research?

The World Health Organization (WHO) has set a goal of reducing tuberculosis deaths by 90% by 2030. Still, it is unknown what kinds of immune responses are needed to eliminate the pathogen. Also, conventional antimicrobial treatments for tuberculosis last several months and multidrug-resistant mycobacteria are becoming more and more common. Therefore, new therapeutic and prophylactic treatments are strongly needed. It is likely that different kinds of responses are beneficial in early, established or reactivated disease states. Our current study concentrates on the early responses against mycobacterial infection. Here, we show that innate immune responses can protect against mycobacterial infection, through the increased transcription levels of *macrophage-expressed gene 1* (*mpeg1*), *tumor necrosis factor α* (*tnfα*) and *nitric oxide synthase 2b* (*nos2b*) and downregulation of *superoxide dismutase 2* (*sod2*). Our results can help to give new ideas on how immune mechanisms should be optimally modulated in the early mycobacterial infection.

> **"[…] I am still most amazed by the result that the protective mechanisms in mycobacterial infection operate via the innate immune system in our model."**

What are the main advantages and drawbacks of the model system you have used as it relates to the disease you are investigating?

In this study, we used the zebrafish and *Mycobacterium marinum* to model human tuberculosis. *M. marinum* is a natural pathogen of ectothermic animals and with this model, we are able to follow the natural course of the infection of this host-specific pathogen. *M. marinum* causes a granulomatous disease with necrotic granulomas in adult zebrafish, closely resembling human tuberculosis. Adult zebrafish has both an innate and adaptive immune system, which makes them ideal for immunological studies. Zebrafish also develop fast, produce offspring abundantly, are cheap to maintain and, most importantly, are the least neuro-physiologically developed organisms of the known tuberculosis models. However, zebrafish have anatomical and physiological differences compared to humans

Hanna Luukinen's contact details: Faculty of Medicine and Life Sciences, University of Tampere, Finland.
E-mail: hanna.luukinen@staff.uta.fi

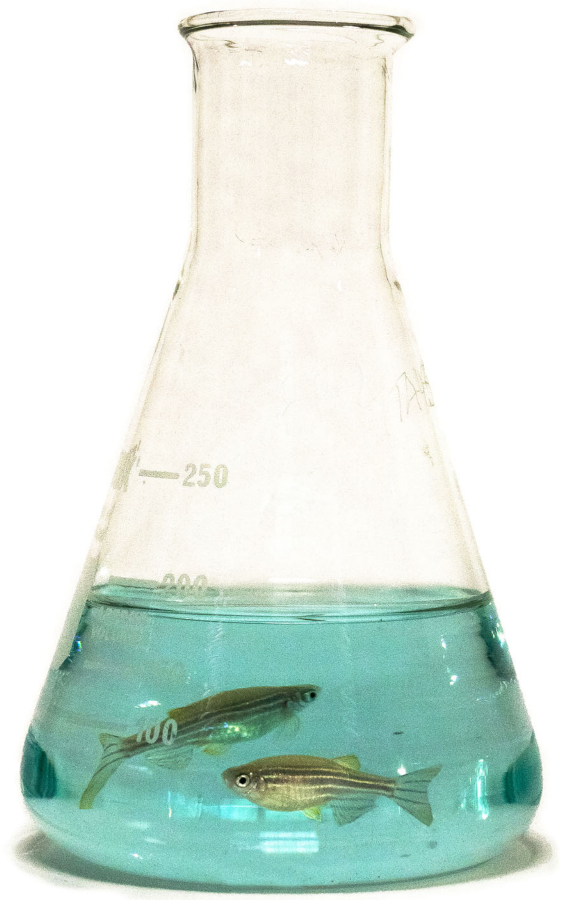

Adult zebrafish used in the research (picture by Lauri Paulamäki).

and lack some commonly used antibody-based techniques, which affected the study design.

What has surprised you the most while conducting your research?

Of all surprises we had during this project, I am still most amazed by the result that the protective mechanisms in mycobacterial infection operate via the innate immune system in our model. How beautiful!

Describe what you think is the most significant challenge impacting your research at this time and how will this be addressed over the next 10 years?

In my opinion, scientists are just scratching the surface of understanding mycobacteria and their different lifestyles. It is a real challenge to fight successfully against this ancient disease. Tuberculosis is mostly seen in developing countries where malnutrition and AIDS are serious problems. However, as people travel around the world actively, I could bet that this disease, including multidrug-resistant forms of mycobacteria, is spreading more broadly. It has been noticed on several occasions that traditional vaccines and treatments are not working perfectly against mycobacteria. If it were easy to protect against tuberculosis, an effective treatment would have already been discovered. I think that the eradication of tuberculosis really requires thinking outside the box and new experimental approaches. Hopefully in 10 years, we have made significant progress.

What's next for you?

The work with these challenging bacteria continues and if I finish my PhD while trying to crack the code, it would be great.

Reference

Luukinen, H., Hammarén, M. M., Vanha-aho, L.-M., Svorjova, A., Kantanen, L., Järvinen, S., Luukinen, B. V., Dufour, E., Rämet, M., Hytönen, V. P. and Parikka, M. (2018). Priming of innate antimycobacterial immunity by heat-killed *Listeria monocytogenes* induces sterilizing response in the adult zebrafish tuberculosis model. *Dis. Model. Mech.* **11**, doi:10.1242/dmm.031658.
